# Properties of the Human Gastric Juice

**Published:** 1877-11

**Authors:** 


### PROPERTIES OF THE HUMAN GASTRIC JUICE.

The *Press and Circular* says M. Charles Ricket has been experimenting upon the patient on whom Prof. Verneuil recently performed the operation of gastrotomy. According to his researches the acidity of the gastric juice is equivalent to 1.7 grammes of hydrochloric acid to 1000 grammes of fluid. This acidity increases a little at the end of digestion. Wine and alcohol also increases it, but cane sugar diminishes it. It tends to return to its normal acidity after the introduction of acid or alkaline matters. The mean duration of digestion is from three to four-anda-half hours, and the food does not pass gradually out of the stomach, but in masses. According to four analyses, after a modification of Schmidt's method, free hydrochloric acid exists in the gastric juice; and altogether this secretion appears to consist of one part of lactic acid to nine parts of hydrochloric acid, the former of which is free in the gastric juice. The nature, therefore, of the free acid in the stomach seems almost solved, and it may be said that in every 1000 grammes of the juice there are 1.53 grains of hydrochloric acid and 0.43 of lactic acid.

